# Hematopoietic stem cell transplantation to improve prognosis in aggressive monomorphic epitheliotropic intestinal T-cell lymphoma

**DOI:** 10.3389/fonc.2024.1388623

**Published:** 2024-11-21

**Authors:** Gi-June Min, Ye Eun Oh, Youngwoo Jeon, Tong Yoon Kim, Byung-Su Kim, Daehun Kwag, Sung-Soo Park, Silvia Park, Jae-Ho Yoon, Sung-Eun Lee, Byung-Sik Cho, Ki-Seong Eom, Yoo-Jin Kim, Seok Lee, Hee-Je Kim, Chang-Ki Min, Jong Wook Lee, Seok-Goo Cho

**Affiliations:** ^1^ Department of Hematology, Seoul St. Mary’s Hematology Hospital, College of Medicine, The Catholic University of Korea, Seoul, Republic of Korea; ^2^ Department of Hematology, Yeouido St. Mary’s Hematology Hospital, College of Medicine, The Catholic University of Korea, Seoul, Republic of Korea; ^3^ Department of Hematology, Eunpyeong St. Mary’s Hematology Hospital, College of Medicine, The Catholic University of Korea, Seoul, Republic of Korea

**Keywords:** monomorphic epitheliotropic intestinal T-cell lymphoma, intestinal perforation, intestinal obstruction, hematopoietic stem cell transplantation, prognosis

## Abstract

**Introduction:**

Monomorphic epitheliotropic intestinal T-cell lymphoma (MEITL) is a rare, aggressive subtype of primary gastrointestinal T-cell lymphoma. Owing to the absence of symptoms characteristic of MEITL, diagnosis can be challenging, and the low response rate to conventional chemotherapy leads to an abysmal prognosis. This study aimed to define the clinicopathologic characteristics of MEITL in Korea, evaluate the clinical outcomes of intensive chemotherapy with and without hematopoietic stem cell transplantation (HSCT), and explore prognostic factors.

**Methods:**

This single-center retrospective study examined the clinical data of 35 patients diagnosed with MEITL at Seoul St. Mary’s Hospital from May 2012 to May 2023.

**Results:**

We included 22 men and 13 women (median age: 59 years; range: 37–79 years). Many patients exhibited acute abdominal pain (n=23, 65.7%) related to bowel perforation (n=21, 60.0%). Most patients (30/35, 85.7%) underwent surgical intervention to diagnose MEITL, whereas only five were diagnosed via endoscopic evaluation. Of the 32 patients receiving first-line therapy, 4 died before assessment, 10 achieved a complete response (CR), 6 had a relapse, and 18 exhibited progressive disease (PD). Seven of 10 patients received upfront HSCT, either autologous (auto-HSCT, n=4) or allogeneic (allo-HSCT, n=3). All four patients on auto-HSCT died after relapse. All three patients who received allo-HSCT maintained a CR by the final follow-up. Three of 6 patients who relapsed and 13 of 18 exhibiting PD received salvage therapy; one patient on salvage auto-HSCT with cytokine-induced killer cell infusion has survived progression free. Salvage allo-HSCT was performed on 6 of 16 patients; among them, 2 achieved a CR, 2 died after relapse, and 2 died owing to septic shock while maintaining a CR. The remaining patients, who received salvage therapy without HSCT, mostly died owing to PD. The median overall survival was 12.1 months, and the median follow-up was 33.2 months. The 1- and 5-year overall survival was 50.9% and 13.3%, respectively.

**Discussion:**

MEITL is an aggressive disease resistant to conventional therapy. Therefore, intensive chemotherapy followed by upfront allo-HSCT should be considered upon diagnosis. These findings underscore the need for novel therapeutic strategies and further investigation into optimizing treatment protocols for MEITL.

## 1 Introduction

Enteropathy-associated T-cell lymphoma (EATL) is a rare condition originating from lymphocytes within the intestinal epithelium. It was previously classified into types 1 and 2, but the 2016 revision of the World Health Organization (WHO) classification redefined type 1 as EATL and type 2 as monomorphic epitheliotropic intestinal T-cell lymphoma (MEITL) (1, 2). EATL is associated with celiac disease and is relatively uncommon in Asia but more prevalent in Western populations. In contrast, MEITL is not associated with celiac disease and is the most prevalent primary epitheliotropic gastrointestinal (GI) T-cell lymphoma in Asia (1). It typically lacks the inflammatory background and clear necrosis exhibited by classic EATL. In this article, we focus on patients with MEITL, previously classified as EATL type 2.

MEITL manifests as a diverse array of nonspecific symptoms, including abdominal pain, diarrhea, weight loss, anorexia, and abdominal distension. Owing to the absence of more specific symptoms, diagnosis can be challenging and is often only accomplished in later stages of the disease (3–5). Moreover, the low response rate of MEITL to conventional chemotherapy contributes to a dismal prognosis, with an average overall survival rate of less than 7 months (3–5). Given the high resistance of MEITL to chemotherapy, additional therapeutic options, such as allogeneic hematopoietic stem cell transplantation (HSCT) or novel agents targeting specific mutations in MEITL, need to be explored (3, 6). In this study, we conducted a retrospective analysis of patients with MEITL in Korea, aiming to define their clinicopathologic features, determine the clinical outcomes of intensive chemotherapy with and without HSCT, and explore prognostic factors.

## 2 Materials and methods

### 2.1 Patient enrollment and evaluations

This single-center study involved the retrospective analysis of clinical and imaging data obtained from 35 patients diagnosed with MEITL at Seoul St. Mary’s Hospital from May 2012 to May 2023. MEITL is defined by the 2016 WHO classification as a distinct subtype of peripheral T-cell lymphoma (PTCL) (1, 2). We comprehensively reviewed the patients’ medical records to collate their demographics, laboratory test results, diagnosis dates, treatment modalities, the last follow-up date, and clinical prognoses. Pathology reports were obtained from two independent expert pathologists who confirmed the diagnosis of MEITL based on the morphologic and immunophenotypic features (1, 2). Staging was performed via a combination of physical examinations, laboratory studies, and radiologic studies, which included computed tomography (CT) of the neck, chest, and abdomen/pelvis, as well as fluorodeoxyglucose-positron emission tomography/CT (FDG-PET/CT). Staging was mainly conducted according to the Lugano classification for GI tract lymphoma (see **Supplementary Table 1**) (7, 8). We also employed the Cotswolds-modified Ann Arbor staging system for lymphomas diagnosed in extranodal organs (see **Supplementary Table 2**) (9). We conducted a bone marrow (BM) biopsy to confirm the involvement of the disease in the BM. Patients were stratified into prognostic groups according to the International Prognostic Index (IPI) and Prognostic Index for T-cell lymphoma (PIT) scores (1, 10, 11).

The study protocol was endorsed by the Institutional Review Board (IRB) and Ethics Committee of the Catholic Medical Center in the Republic of Korea (KC24RISI0598), and it followed the ethical guidelines outlined in the Helsinki Declaration. Given that the study involved a retrospective analysis of de-identified, routinely collected data, the need for informed consent was waived by the IRB at Seoul St. Mary’s Hospital.

### 2.2 Treatment and response evaluation

The first-line chemotherapy regimen is selected by the physician among anthracycline, ifosfamide, and platinum-based regimens, as no consensus exists on the standard treatment for MEITL. CHOP (cyclophosphamide, doxorubicin, vincristine, and prednisolone), CHOEP (CHOP with etoposide), EPOCH (etoposide, doxorubicin, and vincristine mixed into the same infusion bag and administered continuously over 24 hours for a total of 96 hours, followed by an intravenous bolus of cyclophosphamide), or ProMACE/CytaBOM (cyclophosphamide, doxorubicin, etoposide, bleomycin, vincristine, methotrexate, and prednisone) was used as an anthracycline-based chemotherapy regimen in this study. As an ifosfamide-based regimen, SMILE (dexamethasone, methotrexate, ifosfamide, l-asparaginase, and etoposide) or ICE (etoposide, carboplatin, and ifosfamide) were used in this study. In the case of ICE, dexamethasone and l-asparaginase (DL-ICE regimen) were added to augment its efficacy for lymphomas of the aggressive B-cell or NK/T-cell lineage (12–15). DHAP (dexamethasone, high-dose cytarabine, and cisplatin) was used as a platinum-based therapy. After the third and sixth cycles of chemotherapy, we performed therapeutic response evaluation via CT and FDG-PET/CT. Radiologic features were categorized according to the Lugano classification as a complete response (CR), partial response, stable disease (SD), or progressive disease (PD) (8). After achieving a CR, response evaluations were conducted via imaging every 6 months for 5 years. If patients exhibited PD during first-line therapy, exhibited SD/PD during response evaluation, or relapsed after achieving CR, they underwent salvage therapy with a chemotherapy regimen differing from the first.

### 2.3 Transplantation protocols

Patients who decided to undergo autologous HSCT (auto-HSCT) received chemo-mobilization and BU-MEL-TT (busulfan, melphalan, and thiotepa) with a 20% dose-reduction compared to the regimen introduced in a previous study (12). Peripheral blood stem cell (PBSC) mobilization was carried out after CR was achieved, followed by a consolidative chemotherapy regimen consisting of high-dose methotrexate (3.5 mg/m^2^) on day 1 and cytarabine (2.0 mg/m^2^, every 12 hours) on days 2 and 3. A 10 µg/kg/day granulocyte colony-stimulating factor injection was administered 24 hours after the end of the chemotherapy regimen infusion. The target dose of CD34+ cells was set to >5.0×10^6^/kg, but a minimum dose of 3.0×10^6^/kg was acceptable for transplantation. Where the peripheral CD34+ cell count was <10 cells/μL, with a collection count of <0.7×10^6^/kg on day 1, plerixafor was injected for mobilization. After PBSC mobilization was completed, patients underwent BU-MEL-TT conditioning, which included busulfan at a dose of 2.4 mg/kg/day for three consecutive days (D-8, D-7, and D-6), melphalan at a dose of 40 mg/m^2^/day for 2 days (D-5 and D-4), and thiotepa at a dose of 200 mg/m^2^/day for 2 consecutive days (D-3 and D-2).

Allogeneic HSCT (allo-HSCT) was performed using a reduced-intensity conditioning regimen consisting of 30 mg/m^2^ of fludarabine for 6 consecutive days and 70 mg/m^2^ of melphalan for 1 day, along with fractionated total body irradiation of 800 cGy, administered in four fractionated doses over 2 days. This unique regimen is employed at our institution to enhance lymphoablative activities (13, 14). For graft-versus-host disease (GVHD) prophylaxis in cases involving human leukocyte antigen well-matched unrelated or mismatched donors, two consecutive days of anti-thymocyte globulin administration at a dose of 1.25 mg/kg/day were included. Our institute’s post-engraftment management strategy for GVHD and infection prophylaxis was previously described (15).

### 2.4 Statistical analysis

Overall survival (OS) and progression-free survival (PFS) were defined as the time from pathologic diagnosis to death or the last follow-up, respectively, and calculated from the pathologic diagnosis until disease progression, relapse after complete remission, or death. Patients who remained disease-free at the time of the last follow-up were censored. OS and PFS were estimated using the Kaplan–Meier method, and the log-rank test was used to compare the groups. Using cumulative incidence estimation, we calculated the cumulative incidence of relapse (CIR) and treatment-related mortality (TRM) and compared the groups by using Gray’s test. We treated death from any cause without relapse and the relapse incidence as competing risks for CIR and TRM calculations, respectively. The incidence of acute and chronic GVHD was also calculated using the cumulative incidence method, with non-GVHD death and disease relapse considered as competing risks. Univariate analysis variables were selected based on currently known factors or potential factors affecting survival outcomes (3, 5). Multivariable analyses were performed using stepwise selection among candidate variables chosen from the univariate analysis and excluding highly correlated variables (3, 5). Demographic and clinical characteristics were analyzed using Student’s t-test and the chi-squared test. R software version 3.4.1 (R Foundation for Statistical Computing, Vienna, Austria) was used for statistical analyses, and a p-value <0.05 was considered statistically significant.

## 3 Results

### 3.1 Patients’ characteristics

Baseline characteristics of the 35 patients are presented in **Table 1**. The 22 male and 13 female patients had a median age of 59 (range, 37–79) years. All patients had GI tract involvement: the involvement in 65.7% (n=23) of cases was restricted to the small bowel, 14.3% (n=5) of cases involved the small and large bowels, 11.4% (n=4) of cases involved the large bowel alone, and 8.6% (n=3) of cases involved the stomach and duodenum. Many patients presented with acute symptoms of abdominal pain (n=23, 65.7%) related to bowel perforation (n=21, 60.0%) or obstruction (n=6, 17.1%). Therefore, most patients (30/35, 85.7%) underwent surgical intervention for diagnosis of MEITL, and only five patients were incidentally diagnosed via endoscopic evaluations. Besides abdominal pain, weight loss, anorexia, bowel distension, and diarrhea, patients also presented with GI bleeding (n=2) owing to lesion-related bowel obstruction. Moreover, about half of the patients (n=18, 51.4%) exhibited B-symptoms (at least one of the following symptoms: high fever, weight loss of 10% or more within the past 6 months, and night sweats) at the initial diagnosis. According to the Lugano staging, 14.3% (n=5) had only GI tract involvement (stage I), 2.9% (n=1) had local lymph node involvement (stage II_1_), 22.9% (n=8) had distant abdominal lymph node involvement (stage II_2_), 2.9% had adjacent organ involvement (stage II_2E_), and 57.1% (n=20) were classified as stage IV, with supra-diaphragmatic lymph node or extranodal involvement, most frequently in the BM (n=9) and lungs (n=7). We further divided the stages as localized (stages I and II_1_) and advanced (stages II_2_, II_2E_, and IV). For risk classification, patients were categorized into two groups by using both the IPI and PIT scoring systems: higher-risk (comprising high to high-intermediate risk) and lower-risk (including low-intermediate to low risk) categories. As a result, 9 and 14 patients were categorized into the higher-risk group via IPI and PIT, respectively.

**Table 1:** Clinical characteristics of patients with monomorphic epitheliotropic intestinal T-cell lymphoma.

Characteristics	Values
**Age (years), median (range)**	59 (37–79)
Age >60	12 (34.3%)
Sex	
Male/Female	22 (62.9%)/13 (37.1%)
Present symptoms	
Abdominal pain	23 (65.7%)
Weight loss	12 (34.3%)
Anorexia	10 (28.6%)
Bowel distension	9 (25.7%)
Diarrhea	6 (17.1%)
Gastrointestinal bleeding	2 (5.7%)
Incidental findings without symptoms	3 (8.6%)
Site of involvement	
Small bowel alone	23 (65.7%)
Small and large bowels	5 (14.3%)
Large bowel alone	4 (11.4%)
Gastro-duodenum	3 (8.6%)
B-symptom*	18 (51.4%)
Acute event at presentation	
Bowel perforation/Bowel obstruction	21 (60.0%)/6 (17.1%)
**Surgical/Endoscopic diagnosis**	30 (85.7%)/5 (14.3%)
Performance status	
0–1/2/3–4	25 (71.4%)/7 (20.0%)/3 (8.6%)
**Lactate dehydrogenase (IU/L), median (range)**	330.0 (126.0–926.0)
Normal	21 (60.0%)
Elevated	14 (40.0%)
Bone marrow involvement	
No	26 (74.3%)
Yes	9 (25.7%)
Lugano stage	
**Localized stage**	6 (17.1%)
Stage I	5 (14.3%)
Stage II_1_ (local nodal involvement, para-intestinal)	1 (2.9%)
**Advanced stage**	29 (82.9%)
Stage II_2_ (distant nodal involvement, intra-abdomen)	8 (22.9%)
Stage II_2_E (adjacent organ involvement)	1 (2.9%)
Stage IV	20 (57.1%)
IPI score	
Low risk	13 (37.1%)
Low-intermediate risk	13 (37.1%)
High-intermediate risk	8 (22.9%)
High risk	1 (2.9%)
PIT score	
Low risk	8 (22.9%)
Low-intermediate risk	13 (37.1%)
High-intermediate risk	10 (28.6%)
High risk	4 (11.4%)

IPI, International Prognostic Index; PIT, Prognostic Index for T-cell lymphoma

*B-symptoms were defined as having at least one of the following symptoms: high fever, weight loss of 10% or more within the past 6 months, and night sweats.

### 3.2 Treatment outcomes

The treatment regimens and outcomes of the patients are summarized in **Table 2**. Among the 35 patients, 3 died due to surgical wound complications and 32 underwent chemotherapy. Various first-line chemotherapy regimens were utilized: 18 anthracycline-based regimens (8 CHOP, 5 EPOCH, 3 ProMACE/CytaBOM, and 2 CHOEP), 13 ifosfamide-based regimens (7 DL-ICE and 6 SMILE), and 1 platinum-based regimen (1 DHAP). Among the 32 patients who received first-line therapy, 4/32 (12.5%) died before assessment, and only 10/32 (31.3%) achieved a CR and 18/32 (56.2%) exhibited PD upon treatment. Among the 10 patients who achieved a CR after first-line treatment, 7 received ifosfamide-based chemotherapy regimens (5 DL-ICE and 2 SMILE), while only 3 patients who received anthracycline-based regimens (2 EPOCH and 1 ProMACE/CytaBOM) achieved CR. Notably, none of the patients who received either CHOP or CHOEP achieved a CR after first-line treatment. One patient died 17 days after the initiation of DHAP regimen due to intraabdominal abscess-related septic shock. Among patients who achieved a CR, 7/10 patients received upfront HSCT [either auto-HSCT (n=4) or allo-HSCT (n=3)] as consolidation. All four patients who underwent auto-HSCT died after relapse, whereas all three patients who received allo-HSCT exhibited a maintained CR at the final follow-up. Among the 10 patients who initially achieved a CR, 6 of them, including 4 who underwent upfront auto-HSCT, experienced relapse. Five of the relapsed patients presented with symptoms, such as sudden bowel perforation (n=3), obstructive ileus (n=1), and hematochezia (n=1), without any evidence of relapse observed in the imaging workup. A relapse was detected via imaging in only one patient, who had no symptoms of relapse.

**Table 2:** Treatment outcomes of patients with monomorphic epitheliotropic intestinal T-cell lymphoma.

Characteristics	Values
**Lines of treatment, median number (range)**	1 (0–4)
First-line treatment*	
No chemotherapy	3/35 (8.6%)
CHOP	8/35 (22.9%)
EPOCH	5/35 (14.3%)
ProMACE/CytaBOM	3/35 (8.6%)
CHOEP	2/35 (5.7%)
SMILE	6/35 (17.1%)
DL-ICE	7/35 (20.0%)
DHAP	1/35 (2.9%)
Response after first-line treatment	
CR/PR	10/35 (28.6%)
SD/PD	18/35 (51.4%)
Death with unknown disease status	7/35 (20.0%)
First-line consolidation	
Upfront autologous HSCT	4/10 (40.0%)
Upfront allogeneic HSCT	3/10 (30.0%)
**Relapse after first-line treatment**	6/10 (60.0%)
**Salvage treatment after relapse**	3/6 (50.0%)
**Salvage treatment after progression**	13/18 (72.2%)
Salvage treatment	
DL-ICE	6/16 (37.5%)
ESHAP	4/16 (25.0%)
SMILE	2/16 (12.5%)
EPOCH	1/16 (6.3%)
ProMACE/CytaBOM	1/16 (6.3%)
IVAM	1/16 (6.3%)
DHAP	1/16 (6.3%)
Salvage consolidation	
Autologous HSCT	1/16 (6.3%)
Allogeneic HSCT	6/16 (37.5%)

CR, complete response; HSCT, hematopoietic stem cell transplantation; PD, progressive disease; PR, partial response; SD, stable disease

*Response evaluation was conducted in three-cycle intervals of chemotherapy. CHOP consists of cyclophosphamide, doxorubicin, vincristine, and prednisone. CHOEP consists of cyclophosphamide, doxorubicin, vincristine, etoposide, and prednisone. DHAP consists of dexamethasone, high-dose cytarabine, and cisplatin. DL-ICE consists of dexamethasone, l-asparaginase, ifosfamide, carboplatin, and etoposide. EPOCH consists of etoposide, prednisone, vincristine, cyclophosphamide, and doxorubicin. ESHAP consists of etoposide, methylprednisolone, high-dose cytarabine, and cisplatin. IVAM consists of ifosfamide, etoposide, cytarabine, and methotrexate. ProMACE-CytaBOM consists of cyclophosphamide, doxorubicin, etoposide, bleomycin, vincristine, methotrexate, and prednisone. SMILE consists of dexamethasone, methotrexate, ifosfamide, l-asparaginase, and etoposide.

Three out of the six patients who relapsed and 13 out of the 18 patients with PD received salvage therapy. The salvage therapies included six DL-ICE, four ESHAP, two SMILE regimens, and one regimen each of EPOCH, ProMACE/CytaBOM, IVAM, and DHAP. One patient achieved a CR and subsequently underwent salvage auto-HSCT with cytokine-induced killer (CIK) cell infusion as a clinical trial. This patient survived progression-free until the last follow-up. Salvage allo-HSCT was performed on 6 of the 16 patients, and among them, 2 achieved a CR and survived progression-free, while 2 died owing to septic shock while still exhibiting a CR and 2 died after relapse. The rest of the patients underwent salvage therapy without HSCT and died mostly from PD.

Among the nine patients who underwent allo-HSCT (3 upfront and 6 as salvage therapy), 4 were diagnosed with acute GVHD, resulting in an overall incidence of 44.4% (95% confidence interval [CI], 11.4–73.9), with a median onset of 32.5 days (range, 27–55). Additionally, 4 patients experienced chronic GVHD, with an incidence of 67.3% (95% CI, 6.7–94.4) and a median onset of 5.9 months (range, 2.3–10.3). Two patients developed overall grade I acute skin (stage 2) GVHD, which subsided with conservative care alone. However, the other two patients had overall grade III acute GVHD, affecting the skin (stage 3) and lower gut (stage 2), and required steroid pulse therapy. All four patients with chronic GVHD had moderate-grade involvement, commonly affecting the oral cavity and eyes. Among them, one patient also had liver involvement (score 2), and another had lung involvement (score 1). One patient exhibited overlap syndrome, presenting both acute skin (stage 2) GVHD and chronic oral (score 1) GVHD. None of the acute or chronic GVHD complications resulted in fatal conditions in this cohort. The demographic characteristics, treatment history, and clinical outcomes of patients treated with either frontline or salvage HSCT are presented in **Table 3**.

**Table 3:** Detailed clinical course of 14 patients with monomorphic epitheliotropic intestinal T-cell lymphoma treated with autologous or allogeneic HSCT.

Patient	Age	Sex	Lugano stage	1^st^ line treatment	1^st^ line treatment response	Salvage treatment	Relapse before HSCT	HSCT type	Clinical outcomes	Follow-up period
**1**	**64**	Male	IV	4 cycles of SMILE	CR	–	CR	Frontline auto-HSCT	Relapse → died owing to PD	12.1 mo
**2**	**50**	Female	IV	7 cycles of ProMACE/CytaBOM	CR	–	CR	Frontline auto-HSCT	Relapse → died owing to PD	18.3 mo
**3**	**56**	Male	II_2_	6 cycles of DL-ICE	CR	–	CR	Frontline auto-HSCT	Relapse → died owing to PD	13.4 mo
**4**	**60**	Male	II_2_	5 cycles of SMILE	CR	–	CR	Frontline auto-HSCT	Relapse → died owing to PD	25.3 mo
**5**	**59**	Male	IV	6 cycles of DL-ICE	CR	–	CR	Frontline allo-HSCT MMUD (1 antigen mismatch)	CR, alive	8.3 mo
**6**	**47**	Female	IV	6 cycles of EPOCH	CR	–	CR	Frontline allo-HSCT MSD	CR, alive	33.2 mo
**7**	**60**	Male	I	6 cycles of DL-ICE	CR	–	CR	Frontline allo-HSCT MUD	CR, alive	61.3 mo
**8**	**37**	Male	IV	6 cycles of EPOCH	SD	3ESHAP	CR	Salvage auto-HSCT*	CR, alive	29.9 mo
**9**	**60**	Male	IV	6 cycles of DL-ICE	CR→ Relapse	4EPOCH	PR	Salvage allo-HSCT FMT (haploidentical)	CR, died owing to septic shock	27.6 mo
**10**	**58**	Male	IV	3 cycles of EPOCH	PD	5ESHAP	PR	Salvage allo-HSCT FMT (haploidentical)	CR, died owing to septic shock	8.5 mo
**11**	**48**	Female	IV	4 cycles of CHOP	PD	3ESHAP→2EPOCH	PR	Salvage allo-HSCT MSD	Relapse → died owing to PD	14.6 mo
**12**	**53**	Female	IV	3 cycles of CHOP	PD	3IVAM→3DHAP	SD	Salvage allo-HSCT MSD	Relapse → died owing to PD	14.5 mo
**13**	**68**	Male	II_2_	6 cycles of ProMACE/CytaBOM	PD	6DL-ICE	CR	Salvage allo-HSCT MSD	CR, alive	17.2 mo
**14**	**49**	Male	IV	4 cycles of ProMACE/CytaBOM	PD	3DL-ICE	PR	Salvage allo-HSCT MUD	CR, alive	139.1 mo

Allo-HSCT, allogeneic hematopoietic stem cell transplantation; auto-HSCT, autologous hematopoietic stem cell transplantation; CR, complete response; FMT, family-mismatched donor transplantation; MSD, matched sibling donor; MUD, matched unrelated donor; MMUD, mismatched unrelated donor; PD, progressive disease; PR, partial response; SD, stable disease

*This patient achieved CR and subsequently underwent salvage auto-HSCT with cytokine-induced killer cell infusion as a clinical trial.

CHOP consists of cyclophosphamide, doxorubicin, vincristine, and prednisone. CHOEP consists of cyclophosphamide, doxorubicin, vincristine, etoposide, and prednisone. DHAP consists of dexamethasone, high-dose cytarabine, and cisplatin. DL-ICE consists of dexamethasone, l-asparaginase, ifosfamide, carboplatin, and etoposide. EPOCH consists of etoposide, prednisone, vincristine, cyclophosphamide, and doxorubicin. ESHAP consists of etoposide, methylprednisolone, high-dose cytarabine, and cisplatin. IVAM consists of ifosfamide, etoposide, cytarabine, and methotrexate. ProMACE-CytaBOM consists of cyclophosphamide, doxorubicin, etoposide, bleomycin, vincristine, methotrexate, and prednisone. SMILE consists of dexamethasone, methotrexate, ifosfamide, l-asparaginase, and etoposide.

### 3.3 Survival outcomes and prognostic factors

**Figure 1:**
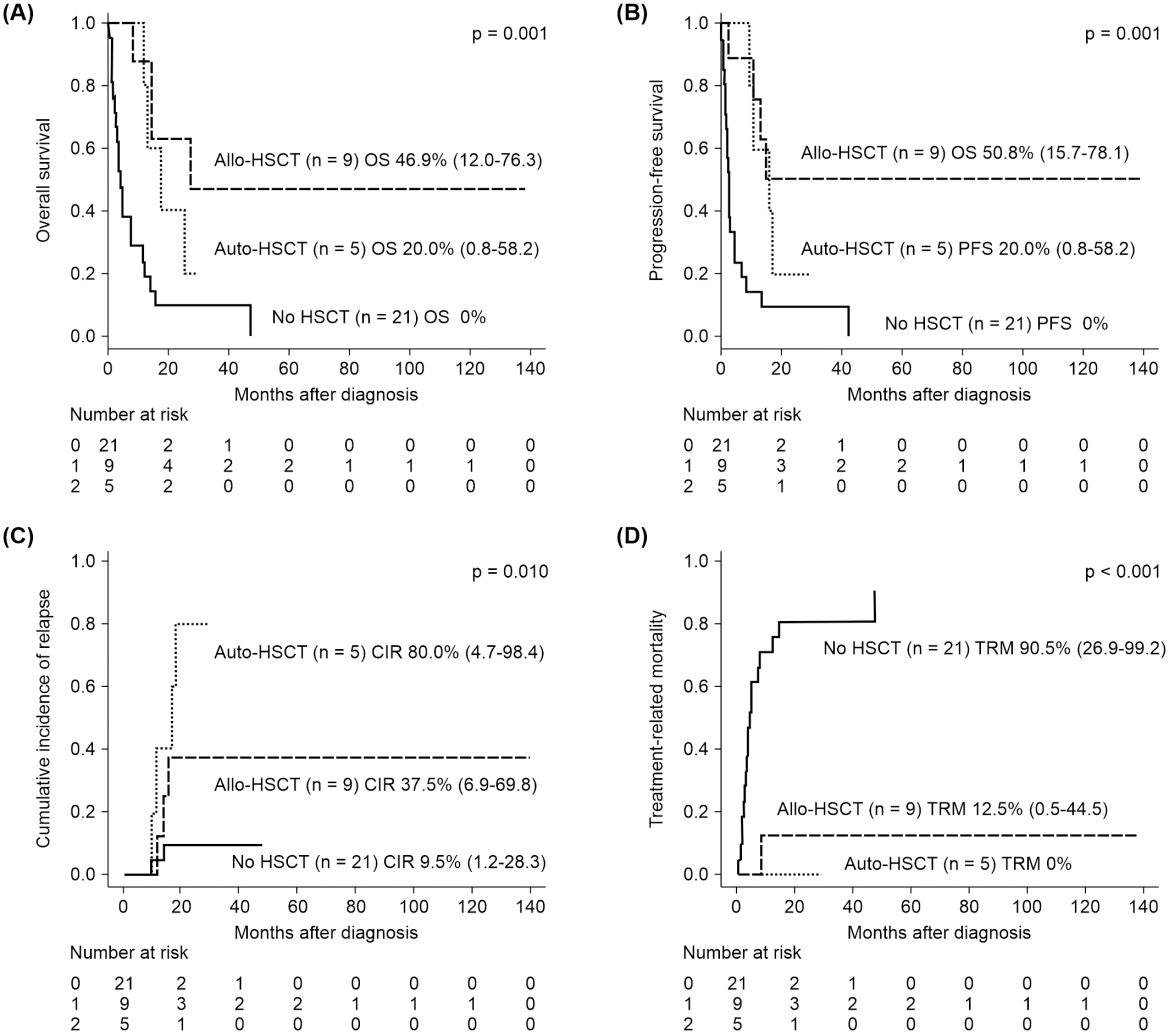
Kaplan–Meier survival curves were used to analyze the treatment outcomes of allo-HSCT, auto-HSCT, and non-HSCT in patients with MEITL. The results revealed significantly better outcomes for patients undergoing allo-HSCT than for patients undergoing other treatments in terms of **(A)** OS and **(B)** PFS. Patients who received auto-HSCT had the highest **(C)** CIR, whereas the no-HSCT group exhibited the highest **(D)** TRM. CIR, cumulative incidence of relapse; HSCT, hematopoietic stem cell transplantation; MEITL, monomorphic epitheliotropic intestinal T-cell lymphoma; OS, overall survival; PFS, progression-free survival; TRM, treatment-related mortality.

The survival outcomes, including OS, PFS, CIR, and TRM, are presented in **Supplementary Figure 1**. After a median follow-up period of 33.2 months, the median OS was 12.1 months. The 1- and 5-year OS were 50.9% and 13.3%, respectively. Additionally, the 1-year PFS, CIR, and TRM were 36.3%, 12.0%, and 46.1%, respectively. However, the 5-year CIR and TRM rates increased to 27.4% and 59.0%, respectively, resulting in a 5-year PFS of 13.8%. Seven of 35 (20.0%) patients achieved a CR, with five of them living beyond 2 years. Six of the seven individuals with a CR had received HSCT (three frontline allo-HSCT, two salvage allo-HSCT, and one salvage auto-HSCT with CIK cell infusion). Therefore, patients who underwent allo-HSCT demonstrated significantly better survival outcomes, including 5-year OS (46.9% vs. 20.0% vs. 0%, p=0.001) and 5-year PFS (50.8% vs. 20.0% vs. 0%, p=0.001) than those who received auto-HSCT or did not undergo any HSCT as a part of their consolidative or salvage treatments. Auto-HSCT recipients had the highest CIR rate, at 80.0%, whereas patients in the non-HSCT group exhibited the highest TRM rate, at 90.5% (**Figure 1**).

**Figure 2:**
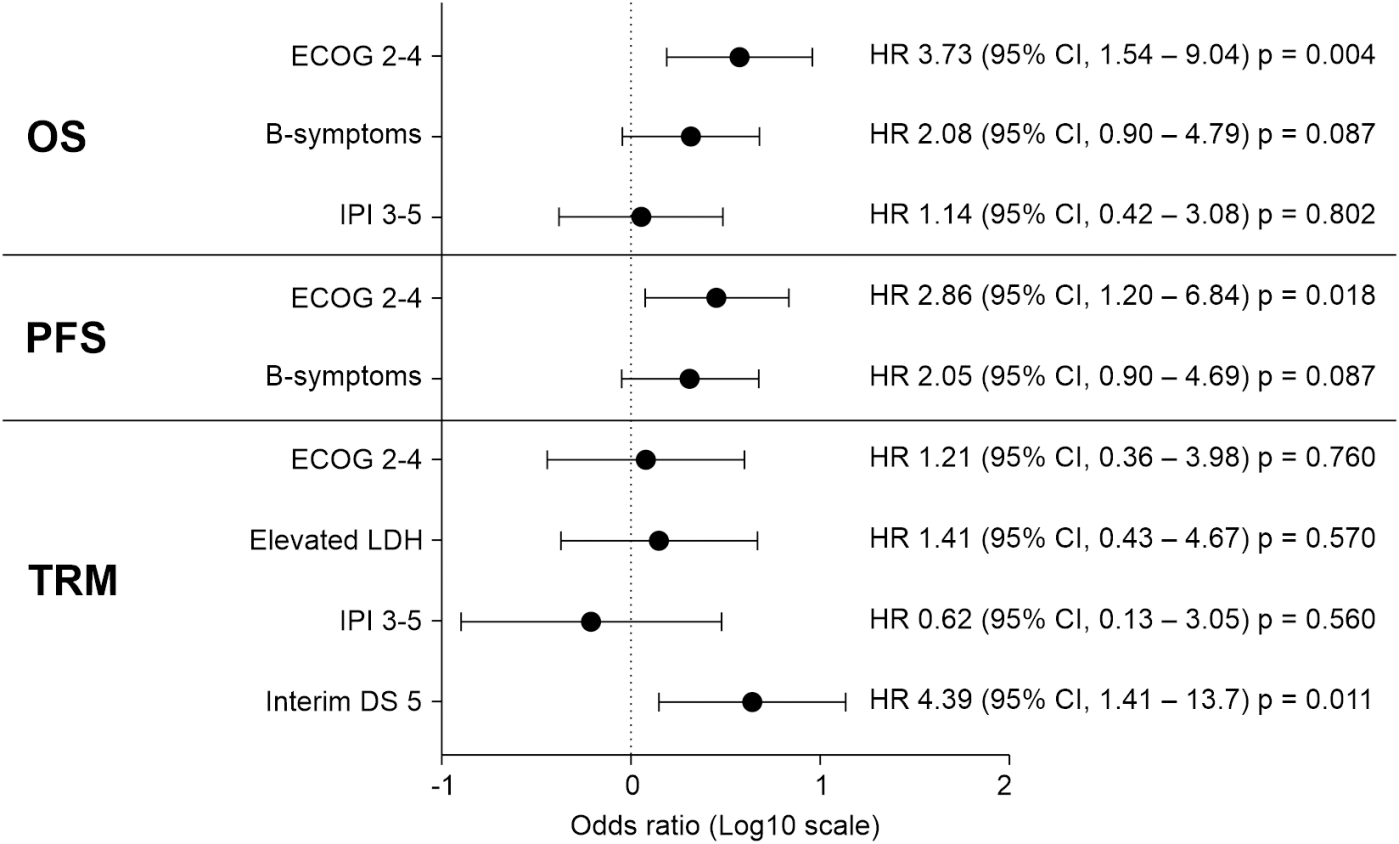
In a multivariable analysis involving 35 patients with MEITL, prognostic factors that had exhibited statistical significance in the univariate analysis were examined using a Cox regression model. The results indicated that an ECOG performance status of 2–4 at the time of diagnosis significantly influenced both OS and PFS. Additionally, an interim FDG-PET/CT DS of 5 was significantly associated with an increased risk of TRM. CI, confidence interval; DS, Deauville score; ECOG, Eastern Cooperative Oncology Group; FDG-PET/CT, fluorodeoxyglucose-positron emission tomography/computed tomography; HR, hazard ratio; IPI, International Prognostic Index; LDH, lactate dehydrogenase; MEITL, monomorphic epitheliotropic intestinal T-cell lymphoma; OS, overall survival; PFS, progression-free survival; TRM, treatment-related mortality.

Prognostic factors exhibiting clinical significance in the univariate analysis were subjected to multivariable analysis by using a Cox regression model (**Figure 2**). As a result, a poor performance status (2 to 4) at the time of diagnosis was significantly associated with a poorer OS (hazard ratio [HR], 3.727; 95% CI, 1.537–9.036; p=0.004) and PFS (HR, 2.862; 95% CI, 1.198–6.840; p=0.018). Additionally, an FDG-PET/CT Deauville score of 5, indicating a mixed response, SD, or PD at the interim response evaluation, was significantly associated with a higher TRM in the multivariable analysis (HR, 4.388; 95% CI, 1.409–13.66, p=0.011). The high-intermediate to high-risk group, classified according to the IPI classification, exhibited significantly poorer OS and TRM in the univariate analysis. However, this significance was not retained in the multivariable analysis. The results of the univariate analysis for prognostic factors are presented in **Supplementary Table 3**.

## 4 Discussion

MEITL is a rare and relatively unknown disease. Therefore, many clinicians and histopathologists are not familiar with or aware of it, and it is sometimes referred to as a “forgotten diagnosis.” (4, 5) The most significant factor contributing to the poor prognosis of MEITL is the delay in recognizing and diagnosing symptoms (3, 4, 16). Frequently, patients have severe symptoms related to GI bleeding, perforation, or obstruction requiring surgical interventions, which are often indicative of disease progression and worsening overall health. Moreover, delayed diagnosis may result in a delay in initiating systemic chemotherapy, and the patient may not have a chance to receive treatment owing to complications. In this study, 60.0% of patients experienced bowel perforation, and 85.7% underwent surgical intervention for the diagnosis of MEITL. To improve the prognosis, clinicians must maintain a high index of suspicion when encountering atypical symptoms, such as unexplained abdominal pain or diarrhea, even in the absence of imaging characteristics or endoscopic evidence of the disease. Awareness of symptoms and maintaining a high index of suspicion are also crucial in detecting relapsed disease in MEITL. In our cohort, the 5-year CIR was 27.4%, but only one patient was diagnosed with relapse during an imaging workup. MEITL relapses often manifest with severe acute symptoms, frequently in the context of surgical treatment indications similar to those observed during the initial diagnosis.

MEITL typically lacks the inflammatory background and clear necrosis observed in classic EATL. Therefore, it is no longer classified as EATL type 2. In terms of immunophenotype, MEITL commonly expresses CD3, CD8, and CD56, although the expression of cytotoxic molecules, such as granzyme B and perforin, varies between patients. All patients in this cohort exhibited CD3, CD8, and CD56 expression without evidence of Epstein–Barr virus (EBV) *in situ*, and despite 31.4% of patients (n=11) exhibiting granzyme B expression, these pathologic results did not significantly influence survival outcomes. Recent investigations have highlighted an increase in *SETD2* mutations and *SYK* expression, suggesting their potential utility in future diagnostic applications (17). Veloza et al. recently published a study on genomic heterogeneity impacting the clinical outcomes of MEITL. They reported that *MYC* expression, *TP53* mutation, and *STAT5B* mutation were strongly associated with poor outcomes, whereas aberrant B-cell marker expression correlated with better survival (3). Approximately 20% of MEITL cases exhibit aberrant expression of CD20 in their study, but none of the patients in our cohort did (3).

The recommended treatment approach for MEITL involves the administration of anthracycline-based chemotherapy with the addition of etoposide, followed by allo-HSCT (5, 18). In our cohort, however, there was a high level of chemoresistance to first-line CHOP-like or anthracycline-based chemotherapy, including CHOP, CHOEP, ProMACE/CytaBOM, and EPOCH, with only 3 of 18 (16.7%) patients achieving a CR. Instead, 7 of 10 patients who achieved a CR after first-line treatment received ifosfamide-based chemotherapy regimens. Furthermore, only 6 out of 18 patients (33.3%) achieved CR during salvage therapy following the failure of first-line treatment. Among these six patients, three underwent DL-ICE, whereas the other three received ESHAP as salvage treatment. Given the aggressive nature of MEITL, a CR status is unlikely to be maintained indefinitely by chemotherapy alone. Auto-HSCT should also be considered along with intensive chemotherapy as consolidation treatment. In fact, the initial treatment strategy in our institute involved either observation or auto-HSCT in the high-risk but tolerable patient groups following the achievement of CR, with subsequent consideration of allo-HSCT in cases of relapse. However, due to the aggressive and rapidly deteriorating nature of MEITL, we have shifted our strategy toward upfront allo-HSCT following the first CR. Therefore, non-CHOP first-line alternatives, such as ifosfamide- or platinum-based chemotherapy, followed by allo-HSCT in eligible patients, could be valuable for the treatment of MEITL (19, 20). In our study, five of seven patients with MEITL who underwent allo-HSCT exhibited a CR up to the last follow-up.

Experts also recommend considering allo-HSCT as the first consolidation treatment for very high-risk diseases, including MEITL (21, 22). Although several cases of upfront auto-HSCT have been presented in clinical trials (20, 23, 24), most enrolled patients were CD30-positive EATL rather than MEITL, with a good overall response rate of 79% (23). However, studies specifically focusing on transplantation treatment for MEITL are extremely rare. Unlike EATL, MEITL typically does not express CD30, making the use of anti-CD30 antibody-drug conjugates challenging. We identified three cases of allo-HSCT for MEITL in the literature, each reported separately, and all cases involved upfront allo-HSCT after achieving remission (3, 5, 25). Two of these patients remained alive without disease relapse (3, 5), while one unfortunately experienced CNS relapse of MEITL and died due to disease progression (25). Given the relatively poor clinical outcomes of upfront auto-HSCT in MEITL observed in our study, upfront allo-HSCT may be considered as an alternative to intensive chemotherapy with or without novel agents, followed by auto-HSCT.

However, the aggressive clinical course and frequent occurrence of severe complications in MEITL, such as bowel perforation and associated septic shock, render many patients ineligible for allo-HSCT. The median post-diagnosis survival is only 7 months, with a 36% OS rate (5), highlighting the poor prognosis of MEITL, which is attributed to chemoresistance associated with driver gene alterations resulting in defective H3K36 trimethylation and dysregulated histone methylation, as well as changes in the JAK/STAT signaling pathways (3). Addressing these unmet clinical needs may be accomplished by using novel agents, such as JAK inhibitors or WEE1 kinase inhibitors, warranting multicenter clinical trials (6, 26).

De Baaij et al. classified patients into high-, intermediate-, and low-risk groups based on B-symptoms and the IPI score, introducing the EATL prognostic index as a predictive tool (27). However, their study excluded patients with MEITL, which may limit its direct applicability to that subgroup. In previous studies, a good performance status and a positive response to initial treatment were significantly associated with better survival outcomes, but age and PIT score were not prognostically significant (3, 5). In our cohort, a poor performance status was significantly associated with a dismal OS and PFS. Additionally, the absence of an interim response, as indicated by an FDG-PET/CT Deauville score of 5, was significantly related to a higher TRM. We also observed that age and PIT score were not significantly associated with survival outcomes. Instead, intermediate-high to high-risk patients, based on the IPI, exhibited significantly poorer OS and TRM in the univariate analysis. However, in the multivariable analysis, this association lost its statistical significance. Therefore, risk prognostication for patients with MEITL and other aggressive PTCL subtypes needs to be improved (28).

This study’s retrospective design and the potential for selection bias due to the limited number of patients and data are substantial limitations. Additionally, relying on data from patients at a single institution and composed of a single racial group may limit the generalizability of the results to only a subset of patients with MEITL. Owing to the rarity of MEITL and of patients with the disease with extended follow-up periods, we were unable to conduct a comprehensive assessment of MEITL-specific genetic mutations, particularly via next-generation sequencing studies. Furthermore, we could not identify variants that express aberrant B-cell markers or detect EBV *in situ* expression, all of which can complicate MEITL diagnosis and its differentiation from other PTCL subtypes (3). The disease resistance to conventional chemotherapy highlights unmet needs in MEITL treatment, necessitating approaches such as allo-HSCT, novel agents, and cell therapies.

In conclusion, MEITL is an aggressive disease with a poor prognosis that is challenging to diagnose, and it exhibits resistance to conventional chemotherapy. Maintaining a high level of suspicion and promptly evaluating patients presenting with unexplained abdominal pain and diarrhea can lead to earlier detection of MEITL, whether in the primary or relapse stage, and enable the initiation of treatment before the development of severe complications, such as bleeding or perforation. This proactive approach has the potential to improve the disease prognosis. We recommend ifosfamide-based chemotherapy followed by allo-HSCT, as chemotherapy alone may not be sufficient to maintain long-term disease remission. Further studies on molecular markers of MEITL and the discovery of pathognomonic mutations may offer insights into the development of novel therapeutic agents to improve the disease prognosis, complementing conventional chemotherapy and transplantation.

## Data Availability

The datasets presented in this article are not readily available because of the nature of this research. Participants of this study disagreed about their data being shared publicly; thus, supporting data are unavailable. Requests to access the datasets should be directed to beichest@nate.com.
