# Supplementary material for: Hematopoietic stem cell transplantation to improve prognosis in aggressive monomorphic epitheliotropic intestinal T-cell lymphoma
Source: Front Oncol. 2024 Nov 21;14:1388623. doi: 10.3389/fonc.2024.1388623 (PMC11617522; doi:10.3389/fonc.2024.1388623)
Supplement: Supplementary file 1 [file DataSheet1.docx]

Supplementary Material

**Supplementary Table 1. Lugano classification for extranodal lymphoma**

| **Classification** | **Features** |
| --- | --- |
| **Localized stage** | |
| **Stage I** | Single primary site or multiple, non-contiguous lesions |
| **Stage II_1_** | Local nodal involvement (para-gastric in cases of gastric lymphoma and para-intestinal for intestinal lymphoma) |
| **Advanced stage** | |
| **Stage II_2_** | Distant nodal involvement (mesenteric in the case of an intestinal primary; otherwise, para-aortic, para-caval, pelvic, and inguinal) |
| **Stage II_2E_** | Enumerate actual site of involvement |
| **Stage IV** | Disseminated extranodal involvement or a gastrointestinal tract lesion with supra-diaphragmatic nodal involvement |

The subscript ‘E’ originally denoted proximal or contiguous, extranodal disease that could be uncompressed within an irradiation field appropriate for nodal disease of the same anatomical extent. The concept of stage III disease within the context of gastrointestinal tract lymphoma was removed altogether, with supra-diaphragmatic nodal disease being included within stage IV.

**Supplementary Table 2. Cotswolds-modified Ann Arbor classification**

| **Classification** | **Features** |
| --- | --- |
| **Localized stage** | |
| **Stage IE** | Single extralymphatic site (IE) or single lymph node region (I) |
| **Stage IIE** | Local extralymphatic extension plus one or more lymph node regions on the same side of the diaphragm (IIE) or two or more lymph node regions on the same side of the diaphragm (II) |
| **Advanced stage** | |
| **Stage III** | Lymph node regions on both sides of the diaphragm (III), which may be accompanied by local extralymphatic extension (IIIE) |
| **Stage IV** | Diffuse involvement of one or more extralymphatic organs or sites |

**Suffix: A**, no B-symptoms; **B**, Presence of at least one of the following: unexplained weight loss of >10% from baseline during the 6 months before staging; recurrent unexplained fever >38°C; recurrent night sweats; **X**, Bulky tumor, defined as either a single mass of tumor tissue exceeding 10 cm in the largest diameter or a mediastinal mass exceeding one-third of the maximum transverse transthoracic diameter measured on a standard posterior-anterior chest radiograph

**Supplementary Table 3. Univariate analysis of survival outcomes in patients with monomorphic epitheliotropic intestinal T-cell lymphoma**

| **Variables** | **OS** | **p-value** | **PFS** | **p-value** | **CIR** | **p-value** | **TRM** | **p-value** |
| --- | --- | --- | --- | --- | --- | --- | --- | --- |
| **Age (years)** |  | 0.822 |  | 0.968 |  | 0.993 |  | 0.916 |
| <60 (n=23) | 18.7% (5.9–37.1) |  | 19.1% (6.1–37.6) |  | 28.1% (10.7–48.6) |  | 53.2% (30.1–71.7) |  |
| ≥60 (n=12) | 0% |  | 0% |  | 25.0% (4.8–53.0) |  | 75.0% (2.1–97.9) |  |
| **Sex** |  | 0.809 |  | 0.896 |  | 0.273 |  | 0.478 |
| Male (n=22) | 17.7% (4.8–37.4) |  | 20.2% (6.5–39.3) |  | 19.74% (5.5–40.2) |  | 60.6% (35.7–78.4) |  |
| Female (n=13) | 0% |  | 0% |  | 38.5% (12.2–64.8) |  | 61.5% (5.3–92.3) |  |
| **ECOG performance status** |  | 0.002 |  | 0.012 |  | 0.112 |  | 0.024 |
| 0–1 (n=25) | 17.8% (4.5–38.9) |  | 18.3% (4.5–39.6) |  | 28.6% (12.0–47.7) |  | 53.1% (26.3–74.1) |  |
| ≥2 (n=10) | 0% |  | 0% |  | 30.0% (1.9–69.5) |  | 70.0% (28.2–90.4) |  |
| **Serum LDH** |  | 0.059 |  | 0.064 |  | 0.184 |  | 0.009 |
| Normal (n=21) | 23.6% (7.8–44.1) |  | 25.6% (9.4–45.7) |  | 35.4% (14.9–56.7) |  | 39.3% (18.2–59.9) |  |
| Elevated (n=14) | 0% |  | 0% |  | 14.3% (1.6–40.2) |  | 85.7% (11.2–98.9) |  |
| **Extranodal involvements** |  | 0.464 |  | 0.489 |  | 0.524 |  | 0.988 |
| 0–1 (n=29) | 11.4% (2.4–28.0) |  | 11.9% (2.6–28.9) |  | 29.9% (13.5–48.4) |  | 58.4% (35.3–75.7) |  |
| ≥2 (n=6) | 33.3% (4.6–67.6) |  | 33.3% (4.6–67.6) |  | 16.7% (0.3–58.5) |  | 50.0% (7.7–82.9) |  |
| **Bone marrow involvement** |  | 0.580 |  | 0.507 |  | 0.953 |  | 0.685 |
| No (n=26) | 8.8% (0.9–28.9) |  | 9.2% (0.9–29.8) |  | 27.7% (11.5–46.7) |  | 63.1% (33.5–82.3) |  |
| Yes (n=9) | 26.7% (4.1–57.9) |  | 27.8% (4.4–59.1) |  | 26.7% (2.4–62.5) |  | 46.7% (11.5–76.5) |  |
| **B-symptoms** |  | 0.013 |  | 0.022 |  | 0.370 |  | 0.151 |
| No (n=17) | 24.8% (5.4–51.4) |  | 26.1% (5.9–53.1) |  | 19.1% (4.2–42.2) |  | 55.4% (20.4–80.3) |  |
| Yes (n=18) | 5.6% (0.4–22.4) |  | 5.6% (0.4–22.4) |  | 33.3% (11.9–56.8) |  | 61.1% (33.9–79.9) |  |
| **IPI risk** |  | 0.035 |  | 0.071 |  | 0.352 |  | 0.016 |
| Low to low-intermediate risk (n=26) | 15.2% (3.5–34.9) |  | 16.0% (3.7–36.1) |  | 33.2% (15.0–52.6) |  | 51.1% (26.2–71.4) |  |
| Intermediate-high to high risk (n=9) | 11.1% (0.6–38.8) |  | 11.1% (0.6–38.8) |  | 11.1% (0.1–48.2) |  | 77.8% (28.1–95.1) |  |
| **PIT risk** |  | 0.140 |  | 0.224 |  | 0.919 |  | 0.162 |
| Low to low-intermediate risk (n=21) | 21.8% (7.1–41.6) |  | 22.9% (7.8–42.5) |  | 29.5% (11.0–50.9) |  | 47.6% (24.9–67.3) |  |
| Intermediate-high to high risk (n=14) | 0% |  | 0% |  | 25.7% (5.1–53.8) |  | 74.3% (12.0–95.9) |  |
| **Lugano stage** |  | 0.777 |  | 0.833 |  | 0.143 |  | 0.361 |
| Stage I to II_1_ (n=6) | 16.7% (0.8–51.7) |  | 16.7% (0.8–51.7) |  | 0% |  | 83.3% (8.6–98.7) |  |
| Stage II_2_, II_2E_, and IV (n=29) | 16.6% (5.5–33.0) |  | 17.7% (6.1–34.1) |  | 33.5% (16.1–52.0) |  | 49.0% (29.4–66.1) |  |
| **Ann-Arbor stage** |  | 0.762 |  | 0.719 |  | 0.669 |  | 0.625 |
| Stage IE and IIE (n=14) | 9.5% (0.6–33.5) |  | 10.7% (0.8–35.4) |  | 21.4% (4.3–47.1) |  | 67.9% (28.3–88.8) |  |
| Stage III and IV (n=21) | 20.5% (6.4–40.1) |  | 20.8% (6.6–40.4) |  | 30.8% (11.7–52.3) |  | 48.7% (25.4–68.6) |  |
| **Bowel perforation as initial diagnosis** |  | 0.940 |  | 0.909 |  | 0.565 |  | 0.715 |
| No (n=14) | 10.7% (0.8–35.4) |  | 10.7% (0.8–35.4) |  | 21.4% (4.4–46.8) |  | 67.9% (28.3–88.8) |  |
| Yes (n=21) | 18.2% (4.8–38.5) |  | 19.6% (5.6–40.0) |  | 32.5% (12.1–54.9) |  | 48.1% (25.1–67.8) |  |
| **Ki-67** |  | 0.458 |  | 0.517 |  | 0.767 |  | 0.600 |
| <80% (n=14) | 19.8% (3.4–46.0) |  | 25.0% (6.3–49.9) |  | 23.8% (4.7–51.0) |  | 52.4% (21.9–75.9) |  |
| ≥80% (n=21) | 0% |  | 0% |  | 28.6% (10.8–49.4) |  | 71.4% (13.8–94.5) |  |
| **Granzyme B** |  | 0.673 |  | 0.881 |  | 0.378 |  | 0.318 |
| Negative (n=24) | 10.6% (1.0–33.4) |  | 11.3% (1.1–34.8) |  | 31.0% (13.1–50.9) |  | 57.9% (26.1–80.1) |  |
| Positive (n=11) | 18.2% (2.9–44.2) |  | 18.2% (2.9–44.2) |  | 18.2% (1.8–48.3) |  | 63.6% (26.6–85.7) |  |
| **Interim response (N=28*)** |  | 0.079 |  | 0.106 |  | 0.224 |  | *0.016* |
| Deauville score 1–4 (n=17) | 28.7% (9.2–51.9) |  | 30.6% (10.5–53.7) |  | 45.2% (18.7–68.6) |  | 24.7% (7.1–47.7) |  |
| Deauville score 5 (n=11) | 9.1% (0.5–33.3) |  | 9.1% (0.5–33.3) |  | 18.2% (1.8–48.3) |  | 72.7% (29.4–92.0) |  |

CIR, cumulative incidence of relapse; ECOG, Eastern Cooperative Oncology Group; IPI, international prognostic index; LDH, lactate dehydrogenase; OS, overall survival; PFS, progression-free survival; PIT, Prognostic Index for T-cell lymphoma; TRM, treatment-related mortality

* Interim response assessment was conducted after the third cycle of chemotherapy using CT and FDG-PET/CT scans.


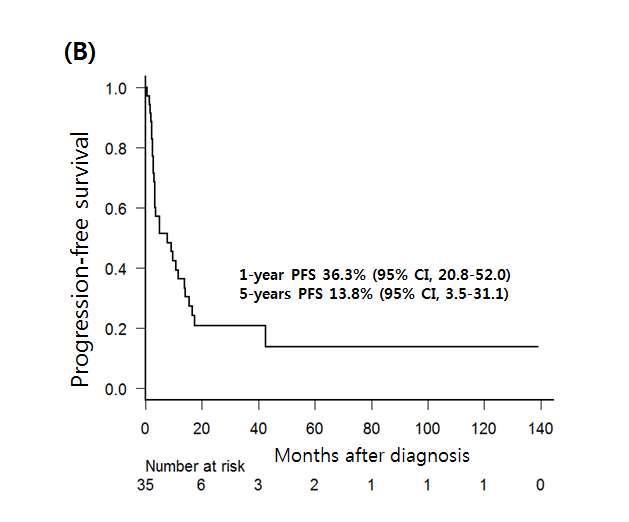

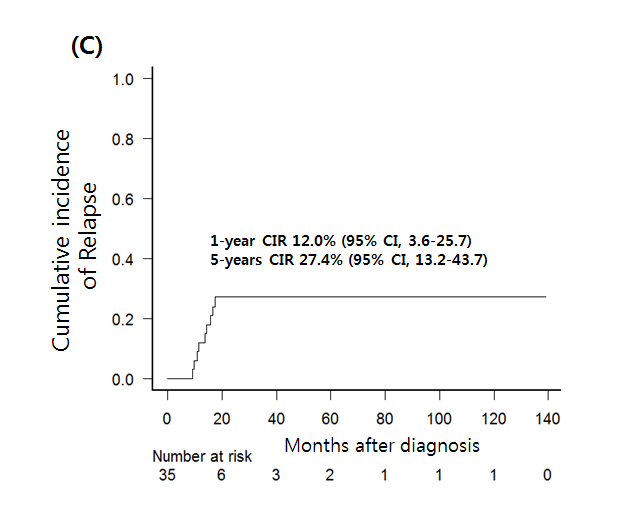

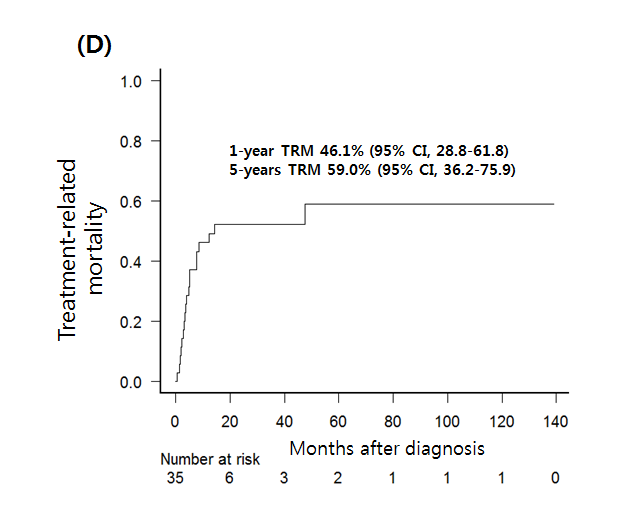

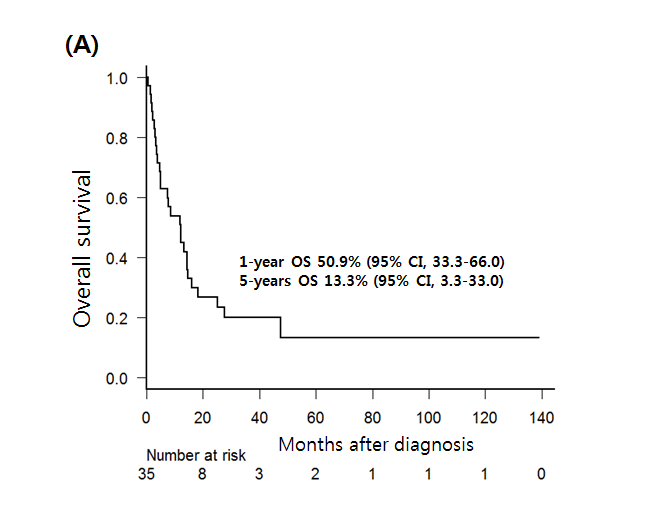


**Supplementary Figure 1.** Survival outcomes of patients with MEITL. The estimated 1- and 5-year OS were (A) 50.9% (95% CI, 33.3%–66.0%) and 13.3% (95% CI, 3.3%–33.0%), respectively. Similarly, the estimated 1- and 5-year PFS were (B) 36.3% (95% CI, 20.8%–52.0%) and 13.8% (95% CI, 3.5%–31.1%), respectively. At 1 year, we observed a (C) CIR of 12.0% (95% CI, 3.6%–25.7%) and (D) TRM of 46.1% (95% CI, 28.8%–61.8%). The estimated 5-year CIR and TRM rates were 27.4% (95% CI, 13.2%–43.7%) and 59.0% (95% CI, 36.2%–75.9%), respectively. CI, confidence interval; CIR, cumulative incidence of relapse; MEITL, monomorphic epitheliotropic intestinal T-cell lymphoma; OS, overall survival; PFS, progression-free survival; TRM, treatment-related mortality.
